# Assessment of Biochemical Composition and Antioxidant Properties of Algerian Date Palm (*Phoenix dactylifera* L.) Seed Oil

**DOI:** 10.3390/plants11030381

**Published:** 2022-01-29

**Authors:** Hamza Harkat, Ratiba Bousba, Cinzia Benincasa, Kamel Atrouz, Mine Gültekin-Özgüven, Ümit Altuntaş, Evren Demircan, Hamdy A. Zahran, Beraat Özçelik

**Affiliations:** 1 Department of Biology and Plant Ecology, Faculty of Natural Sciences and Life, Frères, Mentouri University, Constantine 25000, Algeria; hamza_harkat@yahoo.com (H.H.); bousbaratiba@gmail.com (R.B.); 2 Department of Food Engineering, Faculty of Chemical and Metallurgical Engineering, Istanbul Technical University, Istanbul 34469, Turkey; gultekinmi@itu.edu.tr (M.G.-Ö.); ualtuntas@itu.edu.tr (Ü.A.); evrendemircan@itu.edu.tr (E.D.); ozcelik@itu.edu.tr (B.Ö.); 3 CREA Research Centre for Olive, Fruit and Citrus Crops, C.da Li Rocchi, 87036 Rende, Italy; 4 Oils and Fats Department, Food Industries and Nutrition Research Institute, National Research Centre, Cairo 12622, Egypt; hazahran@hotmail.com

**Keywords:** *Phoenix dactylifera* seed oil, phenols, tocopherols, fatty acids, triacylglycerols, antioxidant activity

## Abstract

Date palm (*Phoenix dactylifera* L.) trees are largely cultivated across the Algerian oases; they are principal sources of remuneration and the economic basis for residents of these areas. Date palm fruits are rich sources of essential nutrients, vitamins, minerals, and dietary fibers, with many potential health benefits, yet there are few studies on the chemical composition and biological properties of date palm seed oil. In this study, we present an in-depth characterization of the biochemical composition and antioxidant properties of date palm seed oil (DPSO) produced in Algeria. DPSOs of eight Algerian cultivars, Arechti, Degla-Baida, Deglet-Nour, Ghars, Haloua, Itima, Mech-Degla, and Tentbouchet, were investigated to determine their biochemical compositions and antioxidant properties. The results highlight the potential of DPSO as an alternative food and a natural resource, thanks to several important compounds having high antioxidant capacity. In particular, fatty acids and triacylglycerol (TAGs) analyses showed that oleic (42.74–50.19%), lauric (18.40–22.2%), and myristic (8.83–10.17%) were the major fatty acids, while 1-myristoyl 2-oleoyl 3-linoleoyl glycerol, 1-linolenoyl 2-oleoyl 3-linoleoyl glycerol, 1-2-linolenoyl 3-linoleoyl glycerol, and 1-linolenoyl 2-myristoyl 3-linoleoyl glycerol were the major TAGs. Biophenols and tocopherols analyses revealed the presence of important compounds, such as catechin (22.04–24.92 mg/kg), vanillin (10.67–23.98 mg/kg), and α-tocopherol (443.59 mg/kg), at high remarkable levels. Therefore, a comparison with the literature data concerning other seed oils, including olive oil, confirms that DPSO can be considered a high-quality oil, from a biochemical and biological point of view.

## 1. Introduction

The date palm tree (*Phoenix dactylifera* L., *Arecaceae*) is considered a symbol of life in some arid areas, due to its tolerance to high temperatures, droughts, and salinity, compared to other fruit crop species. It is one of the oldest trees in which humans have benefitted from, and it has been cultivated since ancient times [1]. The date palm is mainly cultivated in the Middle East and in North Africa, with an annual global production of 9.24 million tons (production increased considerably in the last 30 years) [2]. Date palm trees are principal sources of remuneration and the economic basis for people living in Algerian oases. In these areas, more than 13 million date palm trees and 940 varieties have been recorded to date, with a total production of approximately 1.13 million tons [3,4]. The date palm fruit is considered an ideal food that provides a wide range of essential nutrients; it has many potential health benefits, as it is a rich source of secondary metabolites, dietary fibers, certain essential vitamins, and minerals [5,6,7,8,9]. The main phenolic compounds of date fruits are gallic, protocatechuic, p-coumaric, and ferulic acid, as well as some cinnamic acid derivatives [8,9]. These bioactive compounds, which can quench reactive free radicals, such as superoxide radicals, hydroxyl radicals, or hydrogen peroxide, can prevent the oxidation of molecules, such as proteins and lipids [10]. Furthermore, antioxidants play an important role in human health, as they reduce the risk of major chronic health problems, such as different types of cancer, and cardiovascular and neurological disease [11].

Seeds, in general, from the survival perspective of a plant, are the most important components of fruits, which might explain the exceptionally high concentrations of secondary metabolites contained in the seeds compared to the edible fruit [12,13,14,15,16]. However, there is much variation between results, due to differences in genotypic diversity, growing areas, and cultivation practices [9,13,17,18,19].

Date seeds are good sources of oil (5 to 13%), which is rich in phenolic compounds, tocopherols, and phytosterols; the composition of vitamins, minerals, and fatty acids in date palm seed oil make it valuable in food formulations [16,20]. Several researchers have investigated the chemical composition and antioxidant activity of date seed oil [16,21,22,23,24,25,26,27,28,29,30,31,32,33]. The literature data confirm that DPSO is a source of important compounds, which play important roles in reducing the risk of many diseases [34]. Despite the importance of date seeds, there are few reports in the literature on the chemical compositions and biological properties of its derivatives (e.g., they were not yet studied).

The present study evaluates the chemical compositions and antioxidative properties of a date palm derivative by-product—seed oil—from eight different date palm (*Phoenix dactylifera* L.) cultivars: Arechti, Degla-Baida, Deglet-Nour, Ghars, Haloua, Itima, Mech-Degla, and Tentbouchet, spread locally throughout the province of Biskra, Algeria (Ziban oases). To evaluate date seed oil for its potential use, analyses of tocopherols, fatty acids, triacylglycerols (TAGs), single phenols, total phenolic content (TPC), and antioxidant activity were performed via gas chromatography–flame ionization detector (GC–FID), high-performance liquid chromatography (HPLC), and liquid chromatography–electrospray ionization mass spectrometry (LC–ESI–MS/MS).

Our study demonstrates that since date seeds produce an oil rich in bioactive compounds, which are excellent ingredients for the nutraceutical, pharmaceutical, and cosmetic industries, it should not be treated as “simple waste” but as a raw material. The production of seed oil should be considered a new economic resource that could help in the disposal of by-products derived from date processing.

## 2. Results and Discussion

**Figure 1:**
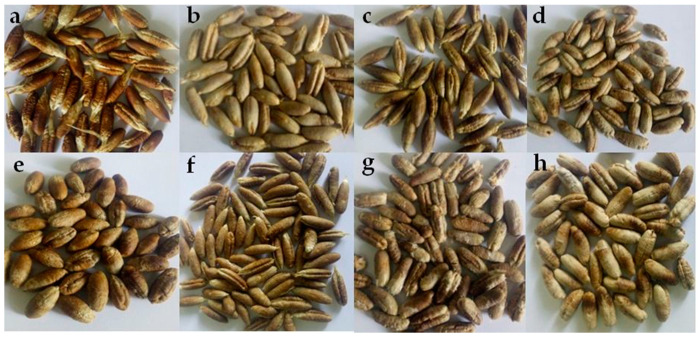
Seeds of different date palm (*Phoenix dactylifera* L.) cultivars. (**a**) Arechti, (**b**) Degla-Baida, (**c**) Deglet-Nour, (**d**) Ghars, (**e**) Haloua, (**f**) Itima, (**g**) Mech-Degla, (**h**) Tentbouchet.

The sampling method for each variety, based on subdividing the palm grove into different plots, was carried out on ten homogeneous trees. Fruit samples were randomly collected from different orientations and heights during the last stage of maturation (in the TMaR stage). The collected fruits, handpicked to eliminate the damaged ones, were pitted to isolate the seeds. The selected seeds were soaked in water, washed to remove any adhering date flesh, and finally dried at 60 °C for 24 h. The dry seeds (Figure 1) were grounded by means of a high-speed mixer under liquid nitrogen, and then lyophilized and stored for less than 30 days at 4 °C before a subsequent analysis.

### 2.1. Oil Extraction Yield

As shown in Table 1, there are significant extraction variation yields of DPSO in between the cultivars: Arechti was the richest in oil, with a total yield of 5.30 g/100 g. This value was lower than the yield obtained by Nehdi et al. [31], who found a value of 7.83 g/100 g in the Barhi cultivar. Degla-Baida was the lowest in oil, with a total yield of 3.41 g/100 g. For the other cultivars, the oil yields were very similar and between 4.52 and 4.93 g/100 g). These values were similar to those indicated by Ali et al. [35]. Many researchers have previously confirmed that DPSO extraction solvents and methods could directly affect the yield extraction as well as the quality of DPSO [35,36,37].

The results clearly demonstrate that date palm seeds can be regarded as important sources of natural oil.

### 2.2. Total Phenolic Content

The total phenolic content (TPC) is vital in defense responses, such as anti-aging, anti-inflammatory, antioxidant, and anti-proliferative activities, through the management of oxidative stress [38]. The results of TPC in the oils analyzed ranged from 154.59 (Deglet-Nour) to 193.35 GAE mg/100 g (Degla-Baida) (Table 1). The total phenol amounts of Algerian DPSO were higher than Moroccan DPSO (181.03 GAE mg/100 g) [32], but lower than Iranian DPSO (1952.93 GAE mg/100 g) [23,33]. The oil extraction process and the solvent used could affect the TPC and antioxidant activity of the oil [26].

### 2.3. Antioxidant Activity

#### 2.3.1. ABTS Radical Scavenging Assay

The results of scavenging activities against the ABTS 2,2′-azinobis (3-ethylbenzothiazoline-6-sulfonic acid) free radical are presented in Table 1 as percent inhibition or µg TE/g, showing that the oil samples under investigation had different scavenging abilities (*p* < 0.05). The seed oil of Degla-Baida, Tentbouchet, and Ghars cultivars had the highest scavenging activity, with 423, 390.5, and 316.5 µg TE/g, respectively, inhibiting 86%, 75.6%, and 74.1% of the ABTS radical, respectively. Deglet-Nour and Arechti oils recorded the lowest activities (50.64 and 40.7%, respectively).

A comparison with extra virgin olive oil, led to similar results, as indicated by Nakbi et al. [39] for Tunisian cultivars (78.56 and 37.23% for Chetoui and Chemlali olive varieties, respectively).

Although there was a relationship between the total amount of phenols and the antiradical activities of the DPSO, this activity may have been partially affected by some other DPSO constituents, such as tocopherols, and some unsaturated fatty acids.

#### 2.3.2. DPPH Radical Scavenging Assay

The DPPH (α,α-diphenyl-β-picrylhydrazyl) free radical scavenging results are presented in Table 1. DPSO samples, except for Deglet-Nour (14.07% corresponding to 102 µg Trolox/g), were characterized by good scavenging activity against DPPH. The most efficient scavenging activity was reported in Degla-Baida (54.17% corresponding to 386 µg TE/g), Ghars, and Tentbouchet (50.56% corresponding to 309 µg TE/g), followed by the cultivars Ghars > Itima > Haloua > Mech-Degla > Arechti > Deglet-Nour.

The results of DPSO scavenging activity against DPPH were higher than those reported for Pistacia lentiscus fruit oil (87 µg TE/g) [40], and for 42 kinds of essential oils (4.29 to 86.66% DPPH inhibition) reported by Lin et al. [41].

The significant higher antioxidant capacity of DPSO can be attributed to its higher content of total phenols and other antioxidant compounds.

### 2.4. Phenols Composition

For the eight cultivars, the phenolic profiles were similar, but their concentrations showed notable variations (Table 2). A total of 12 compounds were determined and quantified. The main phenol found in all DPSO samples was catechin (22.04 to 24.92 mg/kg) followed by vanillin (10.67 to 23.98 mg/kg), vanillic acid (2.04 to 4.94 mg/kg), luteolin (2.76 to 3.45 mg/kg), tyrosol (1.23 to 2.39 mg/kg), and oleuropein (0.37 to 1.38 mg/kg). Homovanillic acid was not found, except in the oil of the Itima cultivar (5.26 mg/kg), whereas caffeic acid, ferulic acid, hydroxytyrosol, luteolin-7-O-glucoside, and luteolin-4-O-glucoside were determined at very low concentrations, but their amounts can still be considered sufficient as bioactive compounds.

A comparison between cultivars showed that Haloua had the highest amount of catechin, vanillic acid, luteolin, and tyrosol. The Mech-Degla cultivar presented the lowest values of vanillin and vanillic acid and tyrosol. The phenols, such as catechin and luteolin in this kind of oil, were never registered before [25,33]. However, the importance of these two phenols have been demonstrated in numerous studies [42,43,44]: catechin works in the prevention of cardiovascular disease and has shown anticancer benefits.

Few of the literature studies have investigated the determination and quantification of DPSO phenols; therefore, the present results are important in clarifying the phenolic compositions and in determining which bioactive compounds are essential and abundant in DPSO.

Many of the previous works [45,46] have shown that a strong relationship exists between the phenol content of oil and its oxidative stability. Hence, phenols can contribute to the oxidative stability of oil, directly or by synergic effects [47].

### 2.5. Fatty Acids Composition

As showed in Table 3, the fatty acid composition of DPSO for the eight cultivars are composed of ten saturated fatty acids and six unsaturated fatty acids. Palmitoleic, heptadecenoic, and eicosenoic acids were not detected in some of them. The fatty acid contents were generally similar for all cultivars. Exceptions were attributed to some fatty acids (C16:1, C17:1, C20:1) not found in some cultivars. However, the major fatty acids were oleic (C18:1ω9), which ranged from 42.74 to 50.19%, followed by lauric (C12:0), from 18.4% to 22.26%, myristic (C14:0), from 8.83% to 10.17%, palmitic acid (C16:0), from 9.11% to 10.37%, linoleic acid (C18:2ω6), from 6.58% to 8.12%, and stearic acid (C18:0), from 3.07% to 3.64%.

The other fatty acids (behenic, lignoceric, linolenic, arachidic, capric, caprylic, margaric, palmitoleic, heptadecenoic, and eicosenoic) were found in small amounts, and less than 1.88%.

Recent studies have shown that the most common monounsaturated fatty acid in the alimentary diet is oleic, which has protective effects against human diseases, in particular cardiovascular and inflammatory diseases, obesity, and some cancers [48,49,50]. The amounts of DPSO fatty acids reported in previous studies [14,27,31,51,52,53] were, in general, very similar to the results obtained here.

A fatty acid analysis showed that DPSO is an important source of saturated (42.97 to 48.39%), unsaturated (51.39 to 57.22%), monounsaturated (43.16 to 50.44%), and polyunsaturated (7.05 to 8.3%) fatty acids.

Oleic/linoleic (O/L) and UFA/SFA ratios ranged from 5.5 (Haloua) to 7.41 (Mech-Degla) and 1.06 (Degla-Baida) to 1.33 (Mech-Degla), respectively. UFA/SFA observed ratios were similar to those reported in Saudi DPSO (1.23 to 1.48) [31]. O/L ratio was much lower in comparison with olive oil, which can range from 3 to 25. UFA/SFA values were also much lower than those of olive oil (4.8) and sunflower oil (6.75) [30,54]. These descriptors, O/L and UFA/SFA, are useful in determining the stability of the oil [54]. Furthermore, linoleic acid content has a great effect on oil oxidation stability [55], meaning that the O/L ratio can be an indicator that describes well the oxidative stability of oils.

Compared to DPSO produced from other varieties cultivated in other countries, the fatty acid profile was relatively similar to DPSO produced in Tunisia, Morocco, Saudi Arabia, UAE, and Sudan [22,26,31,37,51]. However, the DPSO of the varieties cultivated in Iran produced lower levels of oleic (37.60%) and linoleic (6.93%) acids [56]. One possible explanation for the variation of results could be attributed to the pedoclimatic factors impacting the quantitative and qualitative fatty acid compositions.

### 2.6. Tocopherols Composition

Four vitamin E isomers were present in the unsaponifiable fraction of DPSO. As shown in Table 4, the most abundant tocopherol was α-tocopherol, varying from 260.95 to 543.95 mg/kg. Furthermore, β-tocopherol and γ-tocopherol ranged from 32.04 to 89.27 mg/kg and from 45.49 to 113.19 mg/kg, respectively. With regard to δ-tocopherol, the highest value was reported in Haloua seed oil (513.37 mg/kg).

The minimum and maximum of total tocopherol content (TTC) (560.12 and 946.26 mg/kg) were observed in Degla-Baida and Haloua varieties, respectively. Tocopherols, a group of fat-soluble antioxidants, play an important role in health, by protecting fatty acids from harmful oxidation and, in our body, they protect unsaturated fatty acids from oxidation, ensuring the stability of lipid membranes [57].

Plant oils are the richest sources of vitamin E. A comparison between seed oils highlighted that DPSO ranks fourth in terms of tocopherol content, after pomegranate (3483.4 mg/kg), wheat germ (3117.5 mg/kg), and fig (1400.2 mg/kg) seed oils [58]. Although date seeds are abundant, their uses have not been maximized for animal feed. Given the ease of extraction of date seed oil compared to the seeds of the plants mentioned above, it can be considered an important natural source of vitamin E.

Tocopherols, due to their antioxidant characteristics, contribute to many biological functions, such as immune stimulation, anticancer, anti-inflammatory, antidiabetic, and renal activities, and neuro-, cardio-, and hepatoprotective effects [59].

### 2.7. Triacylglycerol Compositions

In vegetable oils, triacylglycerols are the main constituents (99%) and the most important storage lipids [60]. TAG compositions of DPSO samples are summarized in Table 5. A total of 18 TAGs were identified using GC–FID: oils were characterized by the presence of 4 major TAGs (MOL, LaOL, LaLaL, and LaML) and 14 minor TAGs. The most abundant TAGs represent those with medium equivalent carbon number (ECN) ranging from 38 to 44, except for LaLL, LaLaO, MML/LaPL, LaMO, LaOO, MPL/LaSL, and MMO/LaPO. For whole TAGs, the carbon number (CN) ranged from 50 to 54, and ECN ranged from 38 to 48.

Di-unsaturated TAGs represented the most abundant TAG in DPSO (44.20–47.31%), followed by triunsaturated (39.93–44.39%) and disaturated TAGs (11.39–13.28%).

Despite previous researchers [28,29] extracting DPSO, following the same procedure utilized in this work, the results were significantly different. In our study, 17.70% of LaLaL found in Deglet-Nour was higher than the 8.4% reported by Lieb et al. [28] for the same cultivar; however, LaOO was 16.52%, which is higher than the 4.28% reported by Mohd Jaih et al. [29].

This difference in TAG content and percentages can be attributed to several factor, such as the date palm growing area and its genetic background. Subsequently, the “straight” link between TAG and the fatty acid profile showed that the main fatty acids of DPSO were the main monomers of their TAGs: oleic acid was involved in the polymerization of 11 TAGs, and lauric acid in 8.

## 3. Materials and Methods

### 3.1. Plant Material and Sample Preparation

The plant material of this study consisted of date seeds from eight different varieties of date palms (*Phoenix dactylifera* L.), harvested from palm groves in Biskra province (Southeastern Algeria, Arid region). After a preliminary survey with farmers having knowledge about the date palm heritage of this region, and based on the date consistency (dry, soft, or semi-soft), to select some widely-consumed varieties, based on the above, eight varieties were selected from highly representative palm groves spread across the region. Their names were as follows: four soft dates—Deglet-Nour, Ghars, Itima and Tentbouchet; three dry dates—Degla-Baida, Haloua and Mech-Degla; and one semi-soft date—Arechti.

### 3.2. Oil Extraction

The oil extraction from *Phoenix dactylifera* L. seeds was performed as a solid–liquid extraction, depending on the Soxhlet method, by using n-hexane as the extraction solvent. The Soxhlet procedure consisted of placing 30 g of milled date palm seeds inside a thimble loaded into the Soxhlet extraction system (SOXTHERM, Gerhardt, Germany), where 400 mL of n-hexane were refluxed for 3 h over the sample, under increasing temperatures (until 180 °C). After the extraction, the solvent was removed via a rotary vacuum distillation at 40–50 °C. The obtained oil was filtered, kept in a colored bottle, and left at 4 °C until assessment for their phytochemical composition and antioxidant proprieties.

### 3.3. Total Phenolic Content

The amount of TPC in DPSO has been detected by spectrophotometry at 756 nm, according to Folin–Ciocalteu method [61]. For this purpose, a phenolic extraction was previously performed for each sample using a water–methanol solution, according to the COI (COI. 2009) method for olive oil. In general, 2.5 g of sample was dissolved in 7.5 mL methanol/water (80/20, *v*/*v*) and mixed until the mixture was homogenized. The mixture was kept in an ultrasonic bath for 15 min and supernatant was obtained after centrifugation of the mixture for 30 min at 5000 rpm by keeping the temperature at 4 °C. The methanol–water phase containing the phenolic extracts was finally collected. A total of 2.5 mL of x10diluted Folin–Ciocalteu reagent was added to 0.5 mL of phenolic extract, and vigorously vortexed for 3 min. Afterwards, 2 mL of 7.5% sodium carbonate was added, subsequently mixed for 10 sec, and directly incubated for 2 h at room temperature. The TPC was calculated using gallic acid as a reference compound. The results are expressed as mg of gallic acid equivalent per 100 g of oil (GAE/100 g oil).

### 3.4. Anti-Oxidant Activity

#### 3.4.1. ABTS Assay

The ABTS 2,2′-azinobis (3-ethylbenzothiazoline-6-sulfonic acid) assay was used to determine the DPSO radical scavenging power, according to the method described by Pellegreni et al. [62]. A total of 7 and 2.45 Mm of final concentrations of ABTS stock solution and potassium persulfate, respectively, were transformed to a volumetric flask. The solution was left for 16 h in the dark for the formation of ABTS^+^ radical stock solution. Then, an appropriate quantity of ethanol was added to adjust the absorbance of the ABTS^+^ stock solution to 0.700 at 765 nm. The radical scavenging activity of oil against ABTS^+^ was determined after reacting 100 µL of the diluted oil in ethanol, with 2.9 mL of ABTS^+^ solution, measuring the reduction of the absorbance at 765 nm after 6 min. The results are expressed as µg Trolox equivalent per 100 g of oil (µg TE/100 g oil).

#### 3.4.2. DPPH Assay

The free radical scavenging activity of DPSO was determined by a DPPH (α,α-diphenyl-β-picrylhydrazyl) assay based on the method described by Bondet et al. [22]. The absorbance of freshly dissolved DPPH in ethyl acetate was adjusted to 0.700 at 520 nm. A total of 20 mg oil was weighed in a test tube, where 80 μL of ethyl acetate and 2.9 mL of DPPH free radical solution were added. The mixture was vortexed before performing a 30 min of incubation in the darkness at room temperature; absorbance was measured at 520 nm against ethyl acetate. Trolox was used as a reference standard and the results were expressed as µg Trolox equivalent per 100 g of oil (µg TE/100 g oil).

### 3.5. Single Phenols Profile by LC–ESI–MS/MS

Phenolic compounds were extracted from oils as reported by the COI (COI, 2009) method, with slight modifications. In general, 2.5 g of sample were dissolved in 7.5 mL methanol/water (80/20, *v*/*v*) and shaken for 1 min. Afterwards, the mixture was kept in an ultrasonic bath for 15 min and the supernatant was obtained after centrifugation at 5000 rpm/min for 25 min.

An aliquot of the supernatant was taken and filtered through a 5 mL plastic syringe with a 0.45 µm PVDF filter before injection. The determination and quantification of phenols were carried out by LC–ESI–MS/MS, using an MSD SCIEX API 4000 Q-Trap mass spectrometer. The LC–MS was operated in a negative ion mode using multiple reaction monitoring (MRM) with an ion spray voltage (IS) 4500 V, curtain gas 20 psi, temperature 400 °C, ion source gas (1) 35 psi, ion source gas(2) 45 psi, and collision gas thickness (CAD) medium. Entrance potential (EP), declustering potential (DP), entrance collision energy (CE), and exit collision energy (CXP) were optimized for each transition monitored.

### 3.6. Fatty Acids Profile by GC–FID

To identify and quantify fatty acids in DPSO, fatty acid methyl esters (FAMEs) were analyzed using an Agilent 7820A (Agilent Technologies, Inc., Palo Alto, CA, USA) gas chromatography equipped with a flame ionization detector (FID) and a capillary column (30 m × 0.25 mm i.d., 0.25 μm f.t; Agilent 112-8837). The transesterification of fatty acid oil samples to fatty acid methyl esters was performed using the BF3 method [63]. The injector and detector temperatures were maintained at 250 and 280 °C, respectively. The flow rate of carrier gas hydrogen was 40 mL/min, and the split ratio was 1/50. The identification of FAMEs was conducted by comparing the retention time of each FAME against the FAME of the standard mixture.

### 3.7. Tocopherol Profile by HPLC

Tocopherol composition was determined according to the method described by Nehdi et al. [16]. Tocopherol isomers were separated using a normal HPLC system (Agilent, Kyoto, Japan) equipped with an Inertsil ODS-3 normal phase column (250 × 4.6 mm, 5 μm) and SPD-M20A photodiode-array detector. Prior HPLC analysis, 0.2 g of oil was diluted in 2 mL hexane. The identification and quantification of tocopherol isomers were assured by comparing the peak areas with the external standards.

### 3.8. Triacylglycerol Profile by GC–FID

TAG analyses were performed by means of a gas chromatograph (Agilent 7820 A), equipped with a flame ionization detector at 360 °C and a capillary column. The initial temperature was 285 °C for 35 min before increasing at 10 °C/min, to 310 °C, and was kept for 10 min. The carrier gas was helium, and the flow rate was 0.5 mL/min. To ensure homogeneity, 100 mg of oil was melted and vortexed and then dissolved in 10 mL n-heptane (Turkish Food Codex Communique on olive oil and pomace oil, communique number 2010/36). The solution was transferred to a vial for GC analysis. The comparison between the oil’s triacylglycerol retention times and those of the standard TAG mixtures permitted determining the triacylglycerol of DPSO.

### 3.9. Statistical Analysis

The data for each experiment were analyzed using SPSS statistical software version 25. All experiments were performed with two replications and one-way ANOVA; Duncan’s test applied to evaluate differences between mean values (*p* ≤ 0.05).

## 4. Conclusions

Our study provides an in-depth characterization of the biochemical compositions and antioxidant properties of seed oils produced from eight Algerian date palm cultivars. The studied varieties are the most prevalent in the province of Biskra, which represents Algeria’s most productive region regarding the production of dates its by-products. The chromatographic investigation was performed, firstly, for the eight date seed oils, followed by the control of antioxidant proprieties; it turned out to have similar—as well as different—points among them and other vegetable oils. For instance, seed oils of Haloua and Mech-Degla varieties had high levels of tocopherols, which are important for their antioxidant activities, but they also had a high amount of unsaturated fatty acids. Furthermore, the total phenolic content of DPSO was highly correlated to the antioxidant properties, e.g., Degla-Baida seed oil was characterized by high contents of phenolic compounds and TAGs; thus, the highest antioxidant capacity. Haloua seed oil had high levels of major phenols. Haloua, Degla-Baida, and Mech-Degla varieties are agriculturally neglected in their cultivation, and their "spreads” are not considered as economic varieties in terms of consumption. The results reflect that date seed oils possess specific biochemical properties in relation to their high contents of biochemical compounds compared to other more widely consumed varieties. To date, date seeds are generally unexploited and rarely used as animal feed due to their rigidity; investors consider date seeds to be “troublesome waste” that is difficult to isolate and dispose of (to transform to animal feed). On account of the large availability of date seeds, it is necessary to provide a mechanism to facilitate the isolation of seeds for the nutraceutical, pharmaceutical, and cosmetic exploitation of its oil, due to its richness in bio-natural substances, as unsaturated fatty acids, important phenols and, especially, higher amounts of tocopherols. These results highlight the potential of date seed oil as an alternative food and natural resource for several important compounds. Further studies are necessary to confirm these results and to better characterize the potential of this natural oil.

## Figures and Tables

**Table 1:** Antioxidant capacity, TPC, and oil yield of DPSO under investigation.

	Arechti	Degla- Baida	Deglet- Nour	Ghars	Haloua	Itima	Mech-Degla	Tentbouchet
Oil yield (g/100 g)	5.30 ± 0.31 ^d^	3.41 ± 0.27 ^a^	4.93 ± 0.39 ^c^	4.54 ± 0.53 ^b,c^	4.52 ± 0.04 ^b,c^	4.61 ± 0.10 ^b,c^	4.68 ± 0.55 ^b,c^	4.53 ± 0.46 ^b,c^
TPC (mg GAE/100 g)	156.09 ± 02.04 ^c,d^	193.35 ± 05.22 ^b^	154.59 ± 10.02 ^d^	173.19 ± 06.00 ^a^	166.43 ± 09.72 ^c,d^	170.05 ± 16.16 ^c,d^	157.04 ± 03.75 ^c,d^	177.66 ± 09.13 ^b,c^
DPPH (%)	24.96 ± 0.10	54.17 ± 0.56	14.07 ± 0.14	47.10 ± 1.31	41.00 ± 0.28	44.83 ± 0.00	27.22 ± 0.70	50.56 ± 0.42
DPPH (µg TE/g)	138.50 ± 01.41 ^h^	386.00 ± 00.70 ^g^	102.00 ± 03.53 ^f^	292.50 ± 02.82 ^e^	242.5 ± 005.65 ^c^	267.00 ± 02.12 ^d^	226.50 ± 00.00 ^b^	309.00 ± 00.70 ^a^
ABTS (%)	50.64 ± 0.35	86.00 ± 0.42	40.70 ± 1.50	74.1 ± 0.133	70.60 ± 0.07	70.24 ± 0.12	60.08 ± 0.14	75.6v ± 0.50
ABTS (µg TE/g)	215.50 ± 02.82 ^b^	423.00 ± 02.12 ^g^	123.00 ± 00.70 ^a^	316.50 ± 04.24 ^e^	246.50 ± 14.14 ^c^	267.00 ± 04.94 ^d^	220.00 ± 03.53 ^b^	390.50 ± 01.41 ^f^

Note: Different letters in the same row indicate significant differences for *p* < 0.05.

**Table 2:** Phenolic composition of DPSO.

Phenols	Arechti	Degla- Baida	Deglet- Nour	Ghars	Haloua	Itima	Mech-Degla	Tentbouchet
Vanillin	20.13 ± 0.02 ^g^	15.41 ± 0.01 ^d^	14.89 ± 0.04 ^c^	11.77 ± 0.01 ^b^	18.75 ± 0.01 ^f^	17.13 ± 0.00 ^e^	10.67 ± 0.01 ^a^	23.98 ± 0.03 ^h^
Vanillic acid	3.60 ± 0.03 ^e^	03.49 ± 0.0 ^d^	3.73 ± 0.00 ^f^	4.56 ± 0.01 ^g^	4.94 ± 0.02 ^h^	2.75 ± 0.01 ^b^	2.04 ± 0.02 ^a^	2.96 ± 0.01 ^c^
Caffeic acid	0.27 ± 0.01 ^d^	0.24 ± 0.01 ^c^	0.39 ± 0.02 ^f^	0.19 ± 0.01 ^b^	0.22 ± 0.01 ^b,c^	0.14 ± 0.01 ^a^	0.35 ± 0.01 ^e^	0.14 ± 0.01 ^a^
Ferulic acid	0.05 ± 0.01 ^d^	0.04 ± 0.01 ^c,d^	0.03 ± 0.01 ^b,c^	0.31 ± 0.01 ^g^	0.01 ± 0.01 ^a,b^	0.09 ± 0.02 ^e^	ND	0.19 ± 0.01 ^f^
Catechin	23.91 ± 0.04 ^e^	22.04 ± 0.04 ^d^	24.21 ± 0.03 ^a^	20.31 ± 0.04 ^a^	24.92 ± 0.04 ^c,d^	24.34 ± 0.04 ^e^	22.91 ± 0.02 ^b^	24.07 ± 0.03 ^b,c^
Homovanillic acid	ND	ND	ND	ND	ND	5.26 ± 0.11 ^a^	ND	ND
Hydroxytyrosol	0.45 ± 0.03 ^d^	0.34 ± 0.04 ^c^	0.18 ± 0.02 ^b^	0.07 ± 0.02 ^a^	ND	ND	ND	ND
Tyrosol	1.81 ± 0.02 ^e^	1.24 ± 0.01 ^a^	1.7 ± 0.01 ^d^	1.64 ± 0.02 ^c^	2.39 ± 0.01 ^g^	1,5 ± 0.01 ^b^	1.23 ± 0.01 ^a^	2.17 ± 0.01 ^f^
Luteolin	3.06 ± 0.01 ^c^	3.33 ± 0.01 ^e^	3.23 ± 0.01 ^e^	3.14 ± 0.01 ^d^	3.45 ± 0.01 ^g^	2.76 ± 0.01 ^a^	3.35 ± 0.01 ^f^	2.93 ± 0.01 ^b^
Luteolin-7-O-glucoside	0.25 ± 0.01 ^b^	0.17 ± 0.1	ND	ND	ND	ND	ND	ND
Luteolin-4-O-glucoside	0.09 ± 0.01 ^c,d^	0.04 ± 0.02 ^a^	0.1 ± 0.02 ^d^	0.05 ± 0.02 ^a,b^	0.06 ± 0.01 ^a,b^	0.04 ± 0.01 ^a^	0.07 ± 0.01 ^b,c^	0.06 ± 0.01 ^a,b^
Oleuropein	1.38 ± 0.02 ^h^	1.05 ± 0.01 ^f^	1.09 ± 0.01 ^g^	0.88 ± 0.01 ^e^	0.52 ± 0.01 ^b^	0.84 ± 0.01 ^d^	0.65 ± 0.01 ^c^	0.37 ± 0.01 ^a^

Note: Different letters in the same row indicate significant differences for *p* < 0.05. ND: note detected.

**Table 3:** Fatty acid composition (%) in DPSO of eight Algerian cultivars.

Fatty Acid	Arechti	Degla-Baida	Deglet Nour	Ghars	Haloua	Itima	Mech- Degla	Tentbouchet
Caprylic (C8:0)	0.26 ± 0.02 ^b,c^	0.37 ± 0.01 ^f^	0.42 ± 0.02 ^g^	0.31 ± 0.01 ^d^	0.24 ± 0.02 ^b^	0.28 ± 0.02 ^c,d^	0.16 ± 0.01 ^a^	0.35 ± 0.01 ^e^
Capric (C10:0)	0.36 ± 0.01 ^a^	0.45 ± 0.01 ^a^	0.45 ± 0.00 ^a^	0.45 ± 0.01 ^a^	0.33 ± 0.02 ^a^	0.38 ± 0.04 ^a^	0.25 ± 0.02 ^a^	0.61 ± 0.00 ^a^
Lauric (C12:0)	19.76 ± 1.01 ^a,b^	22.26 ± 1.77 ^b^	22.03 ± 0.99 ^b^	20.51 ± 1.19 ^a,b^	20.12 ± 0.86 ^a,b^	21.02 ± 0.85 ^a,b^	18.4 ± 1.25 ^a^	22.19 ± 0.97 ^b^
Myristic (C 14:0)	9.94 ± 0.66 ^a^	10.17 ± 0.97 ^a^	8.83 ± 1.02 ^a^	9.57 ± 0.93 ^a^	10.07 ± 0.72 ^a^	9.84 ± 0.04 ^a^	9.87 ± 0.63 ^a^	10.12 ± 1.19 ^a^
Palmitic (C16:0)	9.38 ± 0.39 ^a,b^	10.37 ± 0.22 ^b^	9.11 ± 0.33 ^a^	9.58 ± 0.08 ^a,b^	9.17 ± 0.70 ^a^	9.82 ± 0.08 ^a,b^	9.74 ± 0.51 ^a,b^	9.78 ± 0.61 ^a,b^
Palmitoleic (C16:1ω7)	0.16 ± 0.01 ^b,c^	ND	ND	0.13 ± 0.00 ^a,b^	ND	0.18 ± 0.04 ^c^	0.13 ± 0.00 ^a,b^	0.12 ± 0.00 ^a^
Margaric (C17:0)	0.19 ± 0.01 ^e^	0.14 ± 0.01 ^d^	0.06 ± 0.01 ^a^	0.06 ± 0.01 ^a^	0.13 ± 0.01 ^c,d^	0.2 ± 0.01 ^e^	0.11 ± 0.01 ^b^	0.12 ± 0.01 ^b,c^
Heptadecenoic (C17:1ω7)	0.13 ± 0.01 ^b^	0.07 ± 0.00 ^a^	0.06 ± 0.01 ^a^	0.11 ± 0.01 ^b^	0.12 ± 0.02 ^b^	0.19 ± 0.03 ^c^	0.12 ± 0.01 ^b^	ND
Stearic (C18:0)	3.52 ± 0.05 ^d,e^	3.59 ± 0.01 ^e^	3.36 ± 0.09 ^c,d^	3.49 ± 0.01 ^c,d,e^	3.64 ± 0.13 ^e^	3.19 ± 0.09 ^a,b^	3.07 ± 0.08 ^a^	3.34 ± 0.06 ^b,c^
Oleic (C18:1ω9)	42.74 ± 1.47 ^a^	43.81 ± 0.04 ^a,b^	46.18 ± 1.49 ^a,b,c^	48.14 ± 1.74 ^c,d^	44.66 ± 0.64 ^a,b^	46.54 ± 1.67 ^b,c^	50.19 ± 1.59 ^d^	44.2 ± 2.06 ^a,b^
Linoleic (C18:2ω6)	6.82 ± 1.01 ^a^	7.45 ± 0.87 ^a^	7.15 ± 0.95 ^a^	6.58 ± 1.12 ^a^	8.12 ± 1.11 ^a^	6.87 ± 1.09 ^a^	6.78 ± 0.87 ^a^	7.89 ± 0.73 ^a^
Linolenic (C18:3ω3)	0.66 ± 0.03 ^d^	0.32 ± 0.01 ^a^	0.69 ± 0.03 ^d^	0.47 ± 0.01 ^b,c^	0.54 ± 0.08 ^c^	0.82 ± 0.10 ^e^	0.47 ± 0.03 ^b,c^	0.41 ± 0.03 ^a,b^
Arachidic (C20:0)	0.59 ± 0.03 ^c,d^	0.33 ± 0.02 ^d^	0.66 ± 0.04 ^d^	0.52 ± 0.03 ^b,c^	0.48 ± 0.02 ^b^	0.77 ± 0.09 ^e^	0.5 ± 0.07 ^b,c^	0.5 ± 0.02 ^b,c^
Eicosenoic (C20:1ω9)	0.13 ± 0.01 ^b^	0.06 ± 0.01 ^a^	ND	ND	ND	ND	ND	ND
Behenic (C22:0)	1.88 ± 0.02 ^e^	0.22 ± 0.02 ^b^	0.59 ± 0.03 ^d^	0.41 ± 0.03 ^c^	0.31 ± 0.01 ^b^	0.43 ± 0.01 ^c^	0.32 ± 0.02 ^b^	0.25 ± 0.01 ^a^
Lignoceric (C24:0)	1.59 ± 0.02 ^e^	0.17 ± 0.00 ^a^	0.29 ± 0.02 ^c^	0.25 ± 0.03 ^b^	0.33 ± 0.00 ^d^	0.28 ± 0.02 ^c^	0.24 ± 0.01 ^b^	0.22 ± 0.02 ^b^
SFA	47.47	48.39	46.49	45.62	45.36	46.75	42.97	47.42
UFA	52.73	51.39	53.39	54.96	52.9	53.78	57.22	52.21
O/L (C18:1/C8:2)	6.27	5.89	6.46	7.32	5.5	6.78	7.41	5.61
PUFA	7.48	7.77	7.84	7,05	8.66	7.64	7.25	8.3
MUFA	43.16	43.23	46.24	48.38	44.78	4691	50.44	44.32
UFA/SFA	1.11	1.06	1.15	1.20	1.17	1.15	1.33	1.10

Means ± standard deviations represent fatty acid relative percentages. Superscripts, significant differences (*p* < 0.05). SFA, saturated fatty acid; UFA, unsaturated fatty acid; MUFA, monounsaturated fatty acid; PUFA, polyunsaturated fatty acid; UFA, unsaturated fatty acid.

**Table 4:** Tocopherol compositions of DPSO under investigation.

Tocopherol	Arechti	Degla-Baida	Deglet- Nour	Ghars	Haloua	Itima	Mech-Degla	Tentbouchet
Alpha	310.51 ± 05.65 ^d^	260.95 ± 05.13 ^e^	543.95 ± 11.11 ^a^	432.91 ± 14.78 ^b^	310.86 ± 07.05 ^d^	295.6 ± 10.35 ^d^	443.59 ± 9.64 ^b^	379.86 ± 3.00 ^c^
Beta	89.27 ± 09.46 ^a^	51.6 ± 06.86 ^d^	32.04 ± 7.59 ^e^	54.64 ± 3.69 ^d^	69.18 ± 1.56 ^b,c^	35.82 ± 0.77 ^e^	60.48 ± 08.7 ^c,d,e^	71.68 ± 01.57 ^b^
Gamma	55.36 ± 04.30 ^d,e^	54.45 ± 05.5 ^d,e^	45.49 ± 3.6 ^d^	95.6 ± 13.26 ^b^	52.85 ± 5.72 ^d^	64.25 ± 01.46 ^c^	113.19 ± 1.51 ^a^	86.54 ± 04.48 ^b^
Delta	261.3 ± 05.83 ^d^	193.12 ± 07.3 ^e,f^	257.37 ± 13.77 ^d^	186.01 ± 8.31 ^f^	513.37 ± 11.39 ^a^	414.15 ± 07.53 ^b^	325.09 ± 1.26 ^c^	207.82 ± 7.02 ^e^
TTC	716.44 ^b^	560.12 ^a^	878.85 ^e^	769.16 ^c^	946.26 ^f^	809.82 ^d^	942.35 ^f^	746.02 ^b,c^

Note: Different letters in the same row indicate significant differences, *p* < 0.05.

**Table 5:** TAG composition of DPSO under investigation.

TAG	CN	ECN	Arechti	Degla-Baida	Deglet- Nour	Ghars	Haloua	Itima	Mech-Degla	Tentbouchet
LaLaL	54	38	14.93 ± 0.01 ^d^	14.51 ± 0.20 ^c^	17.70 ± 0.18 ^e^	13.98 ± 0.18 ^a^	14.10 ± 0.00 ^a,b^	14.44 ± 0.07 ^b,c^	14.67 ± 0.25 ^c,d^	14.01 ± 0.12 ^a^
LaLL	54	40	3.07 ± 0.06 ^c^	2.70 ± 0.06 ^a,b^	3.33 ± 0.01 ^d^	2.64 ± 0.01 ^a^	2.99 ± 0.06 ^c^	2.75 ± 0.02 ^b^	2.75 ± 0.02 ^d^	2.61 ± 0.01 ^a^
LaML	50	40	14.66 ± 0.03 ^c^	14.32 ± 0.09 ^b^	14.30 ± 0.08 ^b^	14.23 ± 0.06 ^b^	14.54 ± 0.08 ^c^	13.66 ± 0.05 ^a^	13.77 ± 0.09 ^a^	14.60 ± 0.08 ^c^
LaLaO	54	40	3.19 ± 0.01 ^c^	2.95 ± 0.01 ^a^	3.09 ± 0.01 ^b^	3.00 ± 0.01 ^a^	3.24 ± 0.02 ^c^	3.00 ± 0.02 ^a^	3.03 ± 0.01 ^a,b^	3.86 ± 0.09 ^d^
LaOL	52	42	15.39 ± 0.06 ^d^	15.42 ± 014 ^b^	14.66 ± 0.25 ^a^	15.35 ± 0.14 ^b^	15.43 ± 0.17 ^b^	15.08 ± 0.12 ^b^	15.10 ± 0.10 ^b^	14.65 ± 0.09 ^a^
MML/LaPL	50	42	3.16 ± 0.10 ^c,d^	3.03 ± 0.04 ^b,c^	3.06 ± 0.07 ^b,c^	3.02 ± 0.00 ^b^	3.26 ± 0.00 ^d^	3.07 ± 0.03 ^b,c^	3.15 ± 0.07 ^b,c,d^	2.89 ± 0.05 ^a^
LaMO	50	42	8.17 ± 0.04 ^c,d^	8.25 ± 0.07 ^d^	7.05 ± 0.07 ^a^	7.76 ± 0.05 ^b^	8.07 ± 0.02 ^c,d^	7.81 ± 0.04 ^b^	7.75 ± 0.13 ^b^	7.83 ± 0.05 ^b^
MOL	54	44	14.58 ± 0.00 ^a^	14.25 ± 0.13 ^a^	16.26 ± 0.25 ^d^	15.92 ± 0.09 ^d^	15.01 ± 0.05 ^b^	16.23 ± 0.08 ^d^	16.31 ± 0.03 ^d^	15.54 ± 0.35 ^c^
LaOO	54	44	3.82 ± 0.00 ^b^	3.31 ± 0.03 ^a^	4.20 ± 0.02 ^c^	4.03 ± 0.02 ^b,c^	4.01 ± 0.02 ^b,c^	4.03 ± 0.02 ^b,c^	4.28 ± 0.33 ^c^	3.43 ± 0.28 ^a^
MPL/LaSL	48	44	3.31 ± 0.00 ^b,c^	3.79 ± 0.01 ^d^	2.81 ± 0.02 ^a^	3.28 ± 0.01 ^b^	3.37 ± 0.00 ^b,c^	3.42 ± 0.02 ^c^	3.42 ± 0.02 ^c^	3.70 ± 0.13 ^d^
MMO/LaPO	52/46	44	6.64 ± 0.05 ^d^	6.45 ± 0.00 ^b,c,d^	5.51 ± 0.24 ^a^	6.69 ± 0.03 ^d^	6.51 ± 0.01 ^c,d^	6.40 ± 0.22 ^b,c,d^	6.25 ± 0.01 ^b,c^	6.16 ± 0.04 ^b^
OOL	54	46	1.60 ± 0.02 ^d,e^	1.43 ± 0.02 ^a,b,c^	1.38 ± 0.01 ^a,b^	1.58 ± 0.00 ^d^	1.65 ± 0.01 ^e^	1.54 ± 0.00 ^c,d^	1.51 ± 0.06 ^b,c,d^	1.34 ± 0.14 ^a^
POL	52	46	4.36 ± 0.03 ^b^	5.19 ± 0.13 ^d^	3.92 ± 0.01 ^a^	4.71 ± 0.02 ^c^	4.36 ± 0.02 ^b^	4.77 ± 0.09 ^c^	4.73 ± 0.03 ^c^	5.07 ± 0.08 ^d^
MOO	50	46	1.41 ± 0.05 ^a^	1.36 ± 0.00 ^a^	1.29 ± 0.79 ^a^	1.30 ± 0.00 ^a^	1.31 ± 0.00 ^a^	1.34 ± 0.00 ^a^	1.43 ± 0.12 ^a^	1.33 ± 0.08 ^a^
POO	52	48	2.07 ± 0.07 ^a^	2.95 ± 0.13 ^c^	2.10 ± 0.13 ^a^	2.45 ± 0.05 ^b^	2.09 ± 0.00 ^a^	2.36 ± 0.12 ^b^	2.24 ± 0.05 ^a,b^	2.91 ± 0.02 ^c^
Di-unsaturated TAGs			45.28 ± 0.06 ^b^	46.35 ± 0.02 ^c^	44.20 ± 0.25 ^a^	46.39 ± 0.23 ^c^	45.40 ± 0.17 ^b^	46.21 ± 0.03 ^c^	46.25 ± 0.09 ^c^	47.31 ± 0.09 ^d^
Disaturated TAGs			13.12 ± 0.15 ^c,d,e^	13.28 ± 0.03 ^e^	11.39 ± 0.20 ^a^	13.00 ± 0.04 ^b,c,d,e^	13.15 ± 0.01 ^d,e^	12.90 ± 0.21 ^b,c,d^	12.82 ± 0.10 ^b,c^	12.75 ± 0.13 ^b^
Triunsaturated TAGs			42.03 ± 0.01^d^	40.36 ± 0.06 ^a,b^	44.39 ± 0.45 ^e^	40.60 ± 0.27 ^b^	41.43 ± 0.18 ^c,d^	40.88 ± 0.24 ^b,c^	41.36 ± 0.43 ^c^	39.93 ± 0.04 ^a^

Note: Different letters in the same row indicate significant differences, *p* < 0.5.

## Data Availability

Not applicable.

## Abbreviations

DPSO	date palm seed oil
TPC	total phenolic content
TAGs	triacylglycerols
GAE	gallic acid equivalent
ABTS	2,2′-azinobis (3-ethylbenzothiazoline-6-sulfonic acid)
DPPH	α,α-diphenyl-β-picrylhydrazyl
TE	trolox equivalent
GC–FID	gas chromatography–flame ionization detector
HPLC	high-performance liquid chromatography
LC–ESI–MS/MS	liquid chromatography–electrospray ionization mass spectrometry
O/L	oleic/linoleic
SFA	saturated fatty acids
UFA	unsaturated fatty acids
MUFA	monounsaturated fatty acids
PUFA	polyunsaturated fatty acids
UFA	unsaturated fatty acids
TTC	total tocopherol content
CN	carbon number
ECN	equivalent carbon number
